# From Periodontal Pocket to Systemic Diseases: Mechanistic Insights Into the Role of *Porphyromonas gingivalis* in Host Pathology

**DOI:** 10.1111/jcmm.71327

**Published:** 2026-08-27

**Authors:** Alaa Ahmed Basalim, Emily Ming‐Chieh Lu

**Affiliations:** ^1^ Periodontology Unit, Centre for Host Microbiome Interactions, Faculty of Dentistry, Oral & Craniofacial Sciences King's College London London UK; ^2^ Department of Periodontology, Faculty of Dentistry Taif University Taif Saudi Arabia

**Keywords:** adverse pregnancy outcomes, Alzheimer's disease, cardiovascular disease, colorectal cancer, diabetes, gingipains, lipopolysaccharides, outer membrane vesicles, *Porphyromonas gingivalis*, systemic disease

## Abstract

*Porphyromonas gingivalis* ( *P. gingivalis* ), a keystone pathogen in periodontitis, has been increasingly recognised as a mechanistic bridge linking periodontal infection to pathological destruction in distant organs. *P. gingivalis* virulence factors, including gingipains, lipopolysaccharides (LPS), and outer membrane vesicles (OMVs), mediate complex host–pathogen interactions. In this review, we critically evaluated recent experimental studies which demonstrate the effects of *P. gingivalis* oral infection on systemic diseases, including cardiovascular disease (CVD), diabetes mellitus (DM), adverse pregnancy outcomes (APOs), colorectal cancer (CRC), and Alzheimer's disease (AD). In CVD, DM, and AD, *P. gingivalis* gingipains exert proteolytic activity that disrupts key cellular targets, including endothelial adhesion molecules, insulin receptors in insulin‐responsive tissues, and neuronal proteins. In AD, *P. gingivalis* LPS contributes to neuronal damage by inducing tau hyperphosphorylation and synaptic dysfunction. In APO and AD, *P. gingivalis* OMVs play a central role in compromising barrier integrity. These processes converge on five principal pathogenic pathways: (1) barrier and structural disruption, (2) immune activation and subversion, (3) mitochondrial dysfunction and oxidative stress induction, (4) systemic inflammation, and (5) metabolism‐mediated effects. Understanding these shared pathways underscores the importance of controlling periodontal disease in promoting systemic health.

## 1 Introduction

Periodontitis is a chronic inflammatory non‐communicable disease characterised by a dysbiotic dental biofilm that elicits a sustained, exaggerated host inflammatory response, leading to irreversible loss of periodontal attachment and eventual tooth loss [1]. Beyond its local tissue destruction, periodontitis has been consistently associated with the pathogenesis of several systemic diseases through the haematogenous dissemination of periodontal pathogens and their virulence factors. Among these, *Porphyromonas gingivalis* ( *P. gingivalis* ) has been detected in atheromatous plaques [2], pancreatic and islet tissues [3], amniotic fluid [4], and brain samples from patients with Alzheimer's disease [5].

*P. gingivalis* possesses a broad spectrum of virulence factors that enable it to invade host tissues, manipulate immune responses, and persist within diverse microenvironments. These include gingipains (cysteine proteases), lipopolysaccharides (LPS), fimbriae, and outer membrane vesicles (OMVs), all of which contribute to immune subversion and systemic dissemination [6, 7, 8].

Associations between periodontitis and systemic diseases such as cardiovascular disease (CVD), diabetes mellitus (DM), adverse pregnancy outcomes (APO), Alzheimer's disease (AD), and colorectal cancer (CRC) have been increasingly supported by experimental evidence implicating *P. gingivalis* as a key pathogenic link. This review synthesises recent experimental studies to explore the molecular and cellular pathways through which *P. gingivalis* contributes to systemic pathology. By comparing mechanistic pathogenic pathways among APO, AD, CRC, DM, and CVD, this review provides an integrated framework for understanding how *P. gingivalis* contributes to and promotes the initiation and progression of systemic diseases and for identifying potential shared pathways of its systemic impact.

## 2 Role of *P. gingivalis* in Endothelial and Vascular Damage

Cardiovascular diseases (CVD), including coronary heart disease, cerebrovascular disease, and peripheral artery disease, remain the leading cause of global mortality, with 18 million deaths reported in 2019 [9]. There is a strong, independent association between periodontitis and cardiovascular disease (CVD), supported by compelling epidemiologic, mechanistic, and interventional evidence [10].

*P. gingivalis* promotes thrombosis risk by directly modulating platelet function. In whole blood models, *P. gingivalis* triggered platelet activation and aggregation, leading to increased thrombus formation under high shear conditions [11]. Mechanistically, *P. gingivalis* lipopolysaccharide (LPS) induced pathological changes in the platelet shape, including filopodia formation and spreading via activation of the small GTPase Cdc42—an essential regulator of cytoskeletal remodelling [12]. This morphological transformation was associated with accelerated clot formation and reduced coagulation time [12]. Using both in vitro and in vivo models, it has been demonstrated that *P. gingivalis* invades endothelial cells in a gingipain‐dependent manner, localising near the nucleus within perinuclear vesicles [13]. Critically, *P. gingivalis* infection leads to proteolytic cleavage of key endothelial adhesion molecules, platelet endothelial cell adhesion molecule‐1 (PECAM‐1), vascular endothelial cadherin (VE‐cadherin), and E‐selectin. In zebrafish, this degradation compromises endothelial junction integrity, resulting in increased vascular permeability and systemic vascular leakage [13]. The loss of these adhesion molecules was not observed when using a gingipain‐deficient mutant (ΔK/R‐ab) strain, directly implicating gingipains as the mediators of this process [13].

Furthermore, recent mechanistic insights highlight a mitochondrial pathway through which *P. gingivalis* induces endothelial injury. *P. gingivalis* infection suppresses the expression of Sirtuin 3 (Sirt3), a mitochondrial deacetylase enzyme, in human aortic endothelial cells (HAECs) [14]. Normally, Sirt3 deacetylates cyclophilin D (CypD) to maintain mitochondrial integrity; however, *P. gingivalis* infection disrupts this protective mechanism, leading to CypD hyperacetylation, which results in mitochondrial dysfunction characterised by increased mitochondrial permeability transition pore opening, elevated mitochondrial reactive oxygen species (ROS), and reduced adenosine triphosphate production. This mitochondrial dysfunction is accompanied by enhanced endothelial apoptosis and impaired angiogenic capacity in HAECs [14].

Consistent with these findings, *P. gingivalis* further compromises endothelial homeostasis by disrupting vascular repair mechanisms. In endothelial cells, *P. gingivalis* compromises vascular integrity by suppressing proliferation, inducing endothelial–mesenchymal transition, and promoting apoptosis via Toll like receptor(TLR)/nuclear factor kappa B (NF‐κB) signalling, thereby impairing endothelial repair capacity [15]. Beyond the endothelium, *P. gingivalis* also targets vascular smooth muscle cells (SMCs), inducing apoptosis through TLR2/NF‐κB activation. Apoptotic SMCs release microRNAs (miR‐143/145) within extracellular vesicles. These miRNAs are subsequently internalised by macrophages, where they translocate to the nucleus and transcriptionally upregulate Siglec‐G, an anti‐phagocytic receptor that suppresses efferocytosis [16]. Impaired macrophage efferocytosis reduces the clearance of apoptotic SMCs, leading to the accumulation of cellular debris and promoting the progression of atherosclerotic plaques [16]. *P. gingivalis* further contributes to CVD through its effects on dendritic cells (DCs). Following intracellular invasion, *P. gingivalis* induces a senescent phenotype in DCs, characterised by upregulation of senescence markers and acquisition of a pro‐inflammatory senescence‐associated secretory phenotype [17]. These senescent DCs secrete increased quantities of extracellular vesicles, including exosomes, enriched with inflammatory cytokines (IL‐1β, TNF‐α, IL‐6), senescence‐associated microRNAs, and bacterial components. Importantly, these exosomes propagate senescence and dysfunction in bystander DCs, thereby amplifying systemic inflammation [17].

**FIGURE 1:**
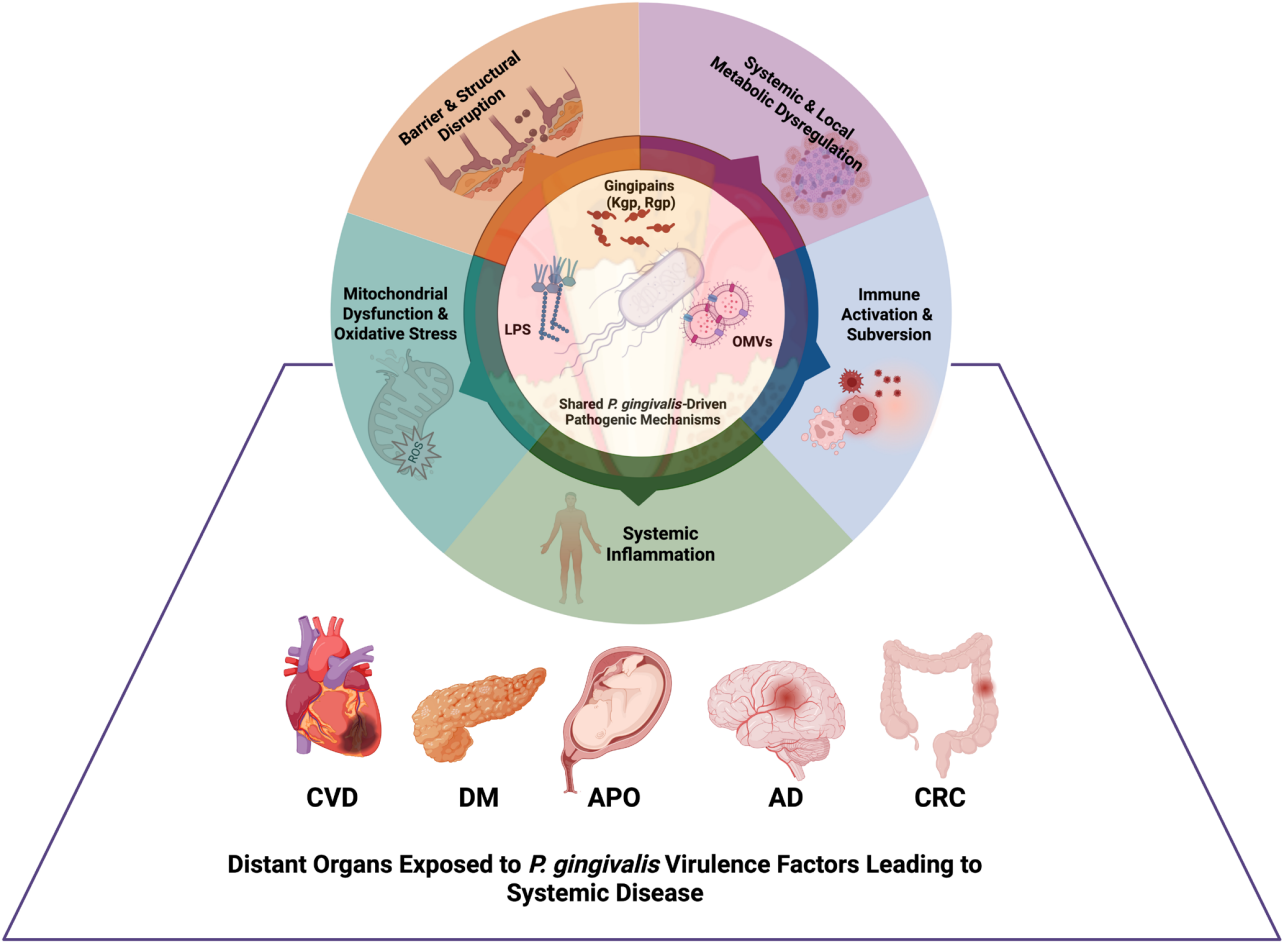
Shared pathogenic mechanisms by which *Porphyromonas gingivalis* drives systemic disease. *P. gingivalis* virulence factors, gingipains, lipopolysaccharides (LPS) and outer membrane vesicles (OMVs) disseminate from periodontal pockets and mediate pathogenic changes in distant organs through five core pathogenic pathways—barrier disruption, immune activation and subversion, mitochondrial dysfunction/oxidative stress, systemic inflammation, and metabolic dysregulation—mediating systemic disease, including cardiovascular disease (CVD), diabetes mellitus (DM), adverse pregnancy outcomes (APO), Alzheimer's disease (AD), and colorectal cancer (CRC). Created in BioRender.com

Together, these experimental studies illustrate how *P. gingivalis* compromises vascular health through multiple mechanisms, including platelet hyperactivation, gingipain‐mediated endothelial adhesion loss, mitochondrial dysfunction, NF‐κB–driven endothelial apoptosis and repair impairment, compromised efferocytosis in vascular SMCs, and DC‐mediated immune subversion (Table 1 and Figure 1).

**TABLE 1:** Virulence factors and mechanisms that associate *P. gingivalis* with different systemic diseases.

Virulence Factor/Mechanism	CVD	DM	APO	AD	CRC
Gingipains	Proteolytic cleavage of key endothelial adhesion molecules; PECAM‐1, VE‐cadherin and E‐selectin [13].	Kgp and Rgp cleave the insulin receptor α subunit in liver, muscles, and adipose tissues [18].		Tau hyperphosphorylation and degradation [5, 19].	
		Mediate AKT/GSK‐3β hepatic insulin signalling impairment and disrupt glycogen synthesis [20].		Amyloid‐β production [5].	
				Synaptic protein loss [5].	
Lipopolysaccharide (LPS)				Activate GSK‐3β leads to tau hyperphosphorylation and synaptic damage [21].	
Outer membrane vesicles (OMVs)			OMV internalised by trophoblasts leads to local metabolic reprogramming, including downregulation of glucose transporter proteins, reduced glycolysis, and impaired cell functions [22].	Compromise BBB by degrading tight junction proteins including claudin‐5, ZO‐1, and occluding [23].	
			OMVs cross the maternal–fetal barrier and directly alter fetal brain development [24].	Tau phosphorylation at threonine‐231 [23].	
				NLRP3 inflammasome activation in microglia [23].	
				Direct neural translocation via trigeminal nerve to the brain [25].	
				Impaired the expression of Brain‐Derived Neurotrophic Factor and *N*‐Methyl‐d‐Aspartate Receptors [25].	
Barrier and structural disruption	Increased vascular permeability via disruption of key endothelial adhesion molecules (PECAM‐1, VE‐cadherin and E‐selectin) [13].	Structural disruption via cleavage of the insulin receptor α subunit in insulin‐target tissues [18].	Reduced trophoblast density [26].	LPS mediated synaptic damage [21].	
	TLR/NF‐κB‐driven endothelial injury and impaired vascular repair [15].	Pancreatic β‐cell transdifferentiation [3].	Impaired spiral artery remodelling [27].	BBB disruption; decreased expression of ZO‐1, claudin‐5, and occluding in brain [23, 28].	
	TLR/NF‐κB‐driven smooth muscle cells apoptosis [16].			Decreased ZO‐1 and occluding in intestine [28].	
				Reduce synaptic and neuronal proteins [28].	
Immune activation and subversion	miRNA‐mediated impairment of macrophage efferocytosis [16].	TLR4/PI3K/AKT pathway activation, mediate by β‐cell inflammation [29].	Reduced uterine natural killer populations and IL‐18 suppression in the placenta [27]	OMV‐induced NLRP3 activation in microglia [23].	Activate deubiquitinase enzyme (UCHL3) that stabilise GNG12 protein which activates the NF‐κB signalling pathway and promote CRC progression [30].
	*P. gingivalis* induces dendritic cell senescence and promotes the release of pro‐inflammatory exosomes that propagate immune dysfunction and amplify systemic inflammation [17].			Neuroinflammatory markers upregulation, ionised calcium‐binding adaptor molecule 1, TLR4, NLRP3, caspase‐1, IL‐1β, IL‐6, TNF‐α in the brain [28].	NLRP3 inflammasome activation in haematopoietic cells [31].
					CHI3L1‐ mediated iNKT‐ cell cytotoxicity dysfunction [32].
Mitochondrial Dysfunction/Oxidative Stress Induction	Sirt3 suppression, leads mitochondrial dysfunction in endothelial cells [14].		Elevated placental oxidative stress, via oxidative marker (Htra1) upregulation [26].		
Systemic inflammation	Immune senescence and exosome‐mediated systemic and vascular inflammation [33].	Elevated levels of circulating pro‐inflammatory cytokines and chemokines in the gingival tissue, serum, liver and adipose tissues associated with downregulation of insulin sensitivity markers [34].		Elevated levels of circulating pro‐inflammatory cytokines and NLRP3 inflammasome activation [25, 35].	
				Gut dysbiosis, brain–gut–microbiota axis [25, 28].	
				Increased intestinal NLRP3, caspase‐1, IL‐1β, IL‐6, TNF‐α [28].	
Systemic and local metabolic dysregulation		Gingipain mediated Akt/GSK3β signalling impairment and glycogen synthesis disruption [20].	OMV‐ induced metabolic reprogramming in trophoblasts, includs downregulation of glucose transporter proteins, reduce glycolysis, and impaired cellular functions [22].		
		Increases circulating BCAA accumulation via the livh/livk system leading to ubiquitination and degradation of Akt2 and subsequently alter glucose metabolism [36, 37, 38].			
		Chronic infection alters hepatic and adipose gene expression, downregulating insulin sensitivity markers and upregulating gluconeogenesis genes, contributing to systemic hyperglycaemia [34].			

*Note:* Summary of key virulence factors and mechanistic pathways identified in recent in vivo and in vitro experiments linking *P. gingivalis* infection to cardiovascular disease (CVD), diabetes mellitus (DM), adverse pregnancy outcomes (APOs), Alzheimer's disease (AD) and colorectal cancer (CRC). Each row outlines a major virulence factor or mechanism implicated in disease‐specific pathophysiology.

Abbreviations: AKT, protein kinase B; BCAAs, branched‐chain amino acids; BBB, blood brain barrier; CHI3L1, chitinase‐3‐like protein 1; CypD, cyclophilin D; GNG12, G protein subunit gamma 12; GSK‐3β, glycogen synthase kinase 3 beta; IL interleukin; iNKT, invariant natural killer; INSR, insulin receptor; Kgp, lysine‐specific gingipain protease; miRNA, microRNA; NF‐κB, nuclear factor kappa‐light‐chain‐enhancer of activated B cells; NLRP3, nucleotide‐binding domain, leucine‐rich–containing family, pyrin domain–containing‐3; PECAM‐1, platelet endothelial cell adhesion molecule 1; PI3K, phosphoinositide 3‐kinase; Rgp, arginine‐specific gingipain protease; TLR, Toll‐like receptor; TNF‐α, tumour necrosis factor alpha; UCHL3, ubiquitin carboxyl‐terminal hydrolase L3; VE‐cadherin, vascular endothelial cadherin; VEGFR1, vascular endothelial growth factor receptor 1; ZO‐1, zonula occludens‐1.

## 3 Role of *P. gingivalis* in Diabetes Mellitus

Diabetes mellitus (DM) is a chronic metabolic disorder characterised by persistent hyperglycaemia due to either autoimmune destruction of pancreatic β‐cells, Type 1 diabetes mellitus (T1DM) or insulin resistance, type 2 diabetes mellitus (T2DM) [39]. While T1DM is primarily an autoimmune condition, emerging evidence highlights the role of low‐grade inflammation in T2DM onset, progression, and complications [40]. The relationship between DM and periodontal disease is well‐established and bi‐directional [41].

Oral *P. gingivalis* infection leads to its translocation to insulin‐sensitive organs, including the liver, muscle, and adipose tissue [18, 20]. In a murine model, repeated exposure to *P. gingivalis* ‐derived outer membrane vesicles (*Pg*‐OMVs) led to elevated fasting blood glucose and impaired insulin responsiveness [20]. Histological and biochemical analyses revealed reduced hepatic glycogen synthesis, which was mechanistically linked to suppression of insulin‐induced phosphorylation of protein kinse B (AKT), a key kinase in the insulin signalling pathway and glycogen synthase kinase‐3β (GSK‐3β), a key mediator of hepatic glucose metabolism [20]. In vitro, HepG2 liver cells exposed to *P. gingivalis* OMVs recapitulated this insulin signalling inhibition in a gingipain‐dependent manner. Importantly, gingipain‐deficient OMVs failed to reproduce these effects, and lipopolysaccharide (LPS) alone did not alter insulin signalling, highlighting gingipain as the key effector responsible for disrupting hepatic glycogen synthesis [20]. Expanding on these findings, Liu et al. (2024) demonstrated a gingipain‐dependent complementary mechanism within the insulin signalling pathway through which *P. gingivalis* can mediate insulin resistance. Specifically, lysine‐specific (Kgp) and arginine‐specific (RgpA/RgpB) gingipains were shown to directly bind to and proteolytically cleave the α subunit of the insulin receptor (INSR) in liver, muscle, and adipose tissues [18]. This cleavage impaired insulin–INSR binding and reduced INSR protein expression, leading to impaired glucose uptake and, therefore, insulin resistance. These effects were verified both in vivo and in vitro and were alleviated in mice infected with gingipain‐deficient *P. gingivalis* strains [18].

Pancreatic islets represent another target of *P. gingivalis* . In both human and murine pancreatic islets, *P. gingivalis* has been detected intra‐ or peri‐nuclearly within β‐cells [3]. Chronic exposure to *P. gingivalis* led to the appearance of bihormonal cells, cells co‐expressing insulin and glucagon, indicating β‐cell transdifferentiation, which may contribute to pancreatic dysfunction in diabetes [3]. Moreover, exposure of rat insulinoma β‐cells and isolated rat islets to supernatants from four different periodontal bacteria, including *P. gingivalis* , led to an increase in inflammatory gene expression up to 5.5‐fold [29]. *P. gingivalis* also induced a significant increase in insulin secretion, up to 5‐fold, mediated via the TLR4/phosphoinositide 3‐kinase (PI3K)/AKT signalling pathway [29]. Importantly, inhibition of TLR4 or PI3K/AKT signalling completely alleviated these effects, suggesting a direct role of *P. gingivalis* virulence factors in promoting β‐cell inflammation and compensatory insulin hypersecretion [29]. Interestingly, infection with *P. gingivalis* mutants that were deficient in branched‐chain amino acid (BCAA) aminotransferase failed to induce metabolic changes, suggesting that *P* *.* *gingivalis* BCAA biosynthesis plays a crucial role in disrupting glycemic control [36]. *P. gingivalis* contributes to increased circulating BCAAs levels in the plasma through its livh/livk system, which encodes components of an ATP‐binding cassette (ABC) transporter involved in BCAA transport. Mice infected with *P. gingivalis* strains lacking livh or livk showed reduced BCAA levels and lower fasting glucose compared to mice infected with wild‐type *P. gingivalis* [37]. Consistent with this, Zhao et al. (2020) demonstrated that elevated systemic BCAAs exacerbate hepatic insulin resistance by activating mechanistic target of rapamycin complex 1 (mTORC1) and suppressing mechanistic target of rapamycin complex 2 (mTORC2) signalling, leading to ubiquitination and degradation of AKT2 [38]. This suppression of AKT2 impaired downstream insulin signalling, reducing lipogenesis and promoting Foxo‐1‐mediated gluconeogenesis, resulting in hyperglycemia and systemic metabolic dysfunction.

Finally, long‐term oral administration of *P. gingivalis* resulted in altered expression of glucose metabolism‐related genes in the liver and adipose tissue [34]. These alterations were associated with low‐grade systemic inflammation, as evidenced by elevated levels of pro‐inflammatory cytokines and chemokines, including tumour necrosis factor alpha (TNF‐α), interleukin‐6 (IL‐6), IL‐17, IL‐23, C–C motif ligand 2 (CCL2), C–C motif ligand 8 (CCL8), and CXCL10 in the gingival tissue, serum, liver and adipose tissues, along with downregulation of insulin sensitivity markers such as insulin receptor substrate 1 and peroxisome proliferator‐activated receptor alpha and upregulation of gluconeogenesis genes such as glucose‐6‐phosphatase, phosphoenolpyruvate carboxykinase 1, and serum amyloid A, which in turn contributed to glucose metabolism impairment in mice [34].

Collectively, experimental studies demonstrate that *P. gingivalis* may contribute to insulin resistance and T2DM through multiple converging pathways: gingipain‐mediated cleavage of the insulin receptor, Akt/GSK‐3β signalling inhibition, β‐cell transdifferentiation, BCAA‐mediated metabolic disruption, and systemic inflammation (Table 1 and Figure 1).

## 4 Role of *P. gingivalis* in Adverse Pregnancy Outcomes

Adverse pregnancy outcomes (APOs) are a broad term for a variety of conditions that include preterm birth, low and very low birth weight, miscarriage, stillbirth, and pre‐eclampsia [42]. The association between periodontal disease and adverse pregnancy outcomes is controversial. While many mechanistic, interventional, and observational studies have suggested a potential link between periodontal pathogens and APOs [43, 44], several methodological shortcomings and inconsistent interventional observations have hindered definitive conclusions [45, 46]. Importantly, there is no evidence to suggest that periodontal therapy improves pregnancy outcomes [47].

Recent studies have shed light on how *P. gingivalis‐*derived vesicles and bacterial by‐products may subtly disrupt placental function and fetal development. One particular insight was demonstrated by an in vitro experimental study, which showed that OMVs released by *P. gingivalis* were internalised by human trophoblast cells via clathrin‐ and caveolin‐mediated endocytosis [22]. Once internalised, the OMVs resulted in alterations in cellular metabolism, including reduced glycolytic activity, reduced expression of glucose transporter proteins (GLUT1 and GLUT3), and reduced reactive oxygen species without overt increase in inflammation. This suggests that the metabolic reprogramming of trophoblasts is mediated by OMV, rather than by inflammation [22]. These metabolic changes significantly compromised trophoblast migration and invasion, both of which are critical functions for satisfactory placental development [22]. The study extended these findings to a mouse model, where early gestational exposure to OMVs led to reduced fetal and placental weights and altered placental GLUT1 expression [22]. In parallel, *Pg*‐OMVs have also been implicated in modulating fetal brain development. A recent in vivo study showed that tail vein injections of *Pg*‐OMVs administered to pregnant C57Bl/6 mice crossed the maternal‐fetal barrier and directly impacted fetal brain development [24]. Specifically, *P. gingivalis* OMV exposure reduced the expression of cortical neuron markers (e.g., Cux1, SatB2) and increased phosphorylation of the Tau protein. Notably, these neurodevelopmental changes occurred without a corresponding rise in cytokine levels in the placental tissue, suggesting a direct, inflammation‐independent mechanism mediated by OMV‐associated virulence factors [24].

Additionally, *P. gingivalis* infection impairs spiral artery remodelling (ISAR) of the uterus and increases placental oxidative stress in genetically susceptible hosts [26]. In a periodontitis model using two different rat strains, Sprague Dawley (SD) and Wistar (WIS) rats, *P. gingivalis* oral inoculation induced ISAR in both. However, only WIS rats developed fetal growth restriction (FGR), despite similar placental bacterial loads. FGR occurring in WIS rats was not associated with overt maternal or placental inflammation, but rather with reduced trophoblast cell density and increased expression of temperature requirement A1 (Htra1), a marker of oxidative stress [26]. Building on these findings, the same group later explored the earlier stages of SAR to expand their mechanistic understanding of *P. gingivalis* ‐mediated placental dysfunction [27]. In this study, intrauterine *P. gingivalis* infection disrupted both the trophoblast‐independent and trophoblast‐dependent phases of spiral artery remodelling. This disruption was mediated by a reduction in uterine natural killer cell populations and interleukin‐18 expression in addition to upregulation of Htra1, linking *P. gingivalis* infection to impaired vascular remodelling and placental oxidative stress, in the absence of systemic or overt maternal inflammation [27].

In summary, *P. gingivalis* and its OMVs can impair trophoblast function, disrupt metabolic homeostasis, and interfere with critical processes such as spiral artery remodelling (SAR). Live *P. gingivalis* can induce host‐mediated processes, including ISAR and oxidative stress, thereby contributing to APOs. Importantly, some of these effects occur in the absence of overt inflammation, suggesting the presence of a *P. gingivalis* ‐mediated APOs pathway involving metabolic reprogramming, oxidative stress, and immune modulation. (Table 1 and Figure 1).

## 5 Role of *P. gingivalis* in Alzheimer's Disease

Alzheimer's disease (AD) is the leading cause of dementia and is marked by gradual cognitive decline alongside key neuropathological features, including amyloid‐β plaque build‐up, tau protein abnormalities, and progressive neurodegeneration [48]. Epidemiological evidence suggests that periodontal disease and immune responses to oral pathogens, particularly *P. gingivalis* , can precede the onset of dementia by several years, with long‐term cohort studies reporting an increased risk of dementia in individuals with periodontitis (hazard ratios generally in the 1.05–2.54 range) [49].

In both in vitro and in vivo models, gingipains induce tau hyperphosphorylation and degradation, promote amyloid‐β production, and cause synaptic protein loss, effects mitigated by specific gingipain inhibitors [5]. Neuronal culture studies demonstrated that gingipains increase the phospho‐tau/tau ratio and reduce synaptic protein levels [19]. Similarly, *P. gingivalis* LPS activated GSK‐ 3β, leading to tau hyperphosphorylation, synaptic damage, and cognitive impairment in AD models [21]. These findings identify gingipains and LPS as key virulence factors in *P. gingivalis* ‐associated neurodegeneration.

*P. gingivalis* OMVs have emerged as key mediators of neuropathology in AD. In an eight‐week oral gavage model, OMVs crossed the blood–brain barrier (BBB), localised in the hippocampus and cortex, and degraded tight junction proteins including claudin‐5, zonula occludens‐1 (ZO‐1), and occludin [23]. This was accompanied by tau phosphorylation at threonine‐231, microglia and astrocytes activation, and IL‐1β levels elevation, which was associated with NLRP3, nucleotide‐binding domain, leucine‐rich–containing family, pyrin domain–containing‐3, inflammasome stimulation in microglia [23]. Pharmacological inhibition of NLRP3 attenuated both inflammatory signalling and tau phosphorylation in neuron–microglia co‐cultures, underscoring its central role in OMV‐mediated neurotoxicity [23]. In a murine bacteremia model, both *P. gingivalis* and its OMVs impaired spatial memory and induced hippocampal inflammation [35]. Interestingly, OMVs largely bypassed systemic immunity compared to whole *P. gingivalis* infection and instead selectively disrupted the BBB, triggering localised neuroinflammation [35]. This supports the notion that OMVs enable *P. gingivalis* to evade systemic immune surveillance while still delivering virulence factors directly to the target tissue. Additionally, *P. gingivalis* ‐derived OMVs, extracted from bacterial cell culture supernatant, have been detected in the trigeminal ganglia and hippocampus [25]. In a murine model, gingival exposure to *P. gingivalis* or its OMVs was taken up by trigeminal nerve endings in the oral cavity and transported to the brainstem and hippocampus, where they activated microglia and induced neuroinflammation, ultimately impairing the expression of Brain‐Derived Neurotrophic Factor and *N*‐Methyl‐d‐Aspartate Receptors [25]. These findings highlight a direct neural conduit through which *P. gingivalis* and its virulence factors can access the central nervous system.

The systemic consequences of chronic *P. gingivalis* infection further support its involvement in AD. Whole bacterium *P. gingivalis* infection elicited a strong systemic inflammatory effect, including weight loss, splenomegaly, elevated circulating pro‐inflammatory cytokines, and NLRP3 inflammasome activation, suggesting an indirect route to neuroinflammation via sustained peripheral immune activation [35]. Complementing these findings, chronic *P. gingivalis* exposure disrupted both intestinal and BBB integrity and altered gut microbiota composition, leading to cognitive deficits [28]. *P. gingivalis* DNA was detected in the brain, along with elevated amyloid‐β precursor protein (AβPP), amyloid‐β fragments, and amyloid‐β₄_2_ (Aβ₄_2_) levels, and tau hyperphosphorylation [28]. Infected mice displayed neuroinflammation in both brain and gut, marked by increased expression of ionised calcium‐binding adaptor molecule 1, NLRP3, caspase‐1, IL‐6, and TNF‐α [28]. These inflammatory changes were associated with structural dysfunction marked by reduced tight junction proteins, ZO‐1 in the brain and ZO‐1 and occluding in the intestine, indicating compromised barrier integrity along with reduced levels of postsynaptic density protein 95, synaptophysin, and the neuronal NeuN proteins [28]. Similarly, gingival but not oral exposure to *P. gingivalis* or its OMVs increased circulating LPS and TNF‐α levels, induced systemic inflammation, and triggered gut microbiota dysbiosis, further implicating a multifactorial brain–gut–microbiota axis [25]. These findings suggest that whole‐bacterium infection induces cognitive impairment not only through direct delivery of virulence factors and barrier disruption but also through microbiota‐driven systemic inflammation.

Collectively, these findings suggest that *P. gingivalis* contributes to AD pathogenesis through multiple complementary pathways: gingipain‐ and LPS‐driven neurotoxicity, OMV‐mediated delivery of virulence factors and NLRP3 activation, extracellular vesicle transport along the trigeminal nerve, and systemic/gut–brain axis inflammation (Table 1 and Figure 1).

## 6 Role of *P. gingivalis* in Colorectal Cancer

Colorectal cancer (CRC) remains a major global health burden, ranking as the third most commonly diagnosed cancer in men and the second in women worldwide [50]. The risk of developing CRC has been shown to increase significantly in the presence of bacterial biofilms within the colonic mucosa, particularly on right‐sided tumours, suggesting a potential pro‐carcinogenic role for these structured microbial communities [51, 52]. Recent evidence suggests that oral pathogens, particularly those associated with periodontitis, can translocate to the colon and contribute to CRC development through mechanisms including biofilm formation, immune modulation, and chronic inflammation [53, 54, 55].

While most mechanistic studies to date have centred on *Fusobacterium nucleatum* [56, 57, 58], growing evidence underscores the emerging significance of *P. gingivalis* in CRC pathogenesis primarily through immune modulation and tumour‐promoting inflammation rather than direct cytotoxicity.

Experimental models have shown that *P. gingivalis* promotes CRC progression by impairing the cytotoxic function of invariant Natural Killer T (iNKT) cells [32]. Mechanistically, *P. gingivalis* induces the expression of chitinase 3‐like 1 (CHI3L1) in iNKT cells, which suppresses their ability to eliminate tumour cells and facilitates immune evasion. Analysis of both patient‐derived samples and murine models revealed that *P. gingivalis* colonisation leads to an increased presence of iNKT cells, a phenotype that is both pro‐inflammatory and immunosuppressive within the tumour microenvironment. In vitro, neutralisation of CHI3L1 restored iNKT cytotoxicity and reactivated signal transducer and activator of transcription 3 (STAT3) signalling, suggesting that CHI3L1 plays a key role in this immune subversion pathway. Importantly, in iNKT cell‐deficient mice, *P. gingivalis* failed to enhance tumour growth, confirming that iNKT cells are essential for its protumor effects and positioning CHI3L1 as a critical mediator of this immune subversion [32].

*P. gingivalis* has also been shown to promote CRC progression through inflammasome activation. In multiple CRC mouse models, *P. gingivalis* exposure significantly increased tumour burden [31]. This effect was driven by activation of the NLRP3 inflammasome in haematopoietic‐derived immune cells. NLRP3 activation led to the establishment of a pro‐inflammatory tumour microenvironment and the recruitment of tumour‐infiltrating myeloid cells. Notably, the tumour‐promoting effects of *P. gingivalis* were abolished in NLRP3‐deficient bone marrow chimeric mice, confirming that haematopoietic NLRP3 signalling is essential for *P. gingivalis* ‐mediated acceleration of CRC progression [31].

Further evidence implicates NF‐κB signalling in *P. gingivalis* ‐driven tumour progression [30]. In both colon cancer cell lines and xenograft models, *P. gingivalis* infection significantly increased the expression of ubiquitin carboxyl‐terminal hydrolase L3 (UCHL3), an enzyme which stabilises its substrate protein, which stabilises Guanine Nucleotide‐Binding Protein Gamma 12(GNG12) through deubiquitination. GNG12, in turn, activates the NF‐κB pathway, leading to enhanced tumorigenic potential and cancer progression [30]. Genetic knockdown of either UCHL3 or GNG12 markedly reduces colorectal cancer cell proliferation and tumour growth [30]. Notably, overexpression of GNG12 in UCHL3‐silenced cells rescued the tumour‐promoting phenotype, underscoring that GNG12 acts downstream of UCHL3 in this signalling pathway. These findings support the critical role of the *P. gingivalis* ‐induced UCHL3–GNG12–NF‐κB axis in driving colorectal cancer progression [30].

Taken together, these findings indicate that *P. gingivalis* contributes to colorectal tumorigenesis primarily by reprogramming the immune microenvironment. Suppression of iNKT cytotoxicity, NLRP3 inflammasome‐driven myeloid recruitment, and NF‐κB pathway activation collectively create a pro‐inflammatory, immune‐suppressed niche that favours tumour growth (Table 1 and Figure 1).

## 7 Discussion

Recent mechanistic in vitro and in vivo studies reinforce the role of *P. gingivalis* in the pathogenesis of multiple distinct systemic diseases through converging pathogenic mechanisms. These include: (1) barrier and structural disruption, (2) immune activation and subversion, (3) mitochondrial dysfunction and oxidative stress induction, (4) systemic inflammation, and (5) metabolism‐mediated effects. Across these interconnected pathways, gingipains, LPS, and OMV act as coordinated virulence factors.

Barrier and structural disruption represent one of the most consistent pathological mechanisms underlying *P. gingivalis* ‐mediated distant tissue damage. Gingipain‐mediated proteolytic cleavage of key endothelial adhesion molecules (PECAM, VE‐cadherin, and E‐selectin) compromises vascular junction integrity and increases vascular permeability, facilitating bacterial dissemination and systemic inflammation [13]. In insulin‐target tissues, *P. gingivalis* gingipains also cleave INSR α subunit, leading to structural and functional disruption of insulin signalling [18], while in pancreatic islets, chronic exposure to *P. gingivalis* induces β‐cell trans‐differentiation, further impairing insulin metabolism [3]. Within reproductive tissues, *P. gingivalis* infection reduced placental trophoblast density and impaired spiral artery remodelling, resulting in APOs [26, 27]. In the central nervous system, OMVs released by *P. gingivalis* compromise the BBB by degrading tight junction proteins, including ZO‐1, claudin‐5, and occludin [23, 28], permitting bacterial components to enter the brain tissues [23, 28]. This BBB dysfunction, together with LPS‐induced tau hyperphosphorylation and synaptic injury [21], contributes to neuroinflammation and neuronal loss in AD. Barrier disruption also occurs in the intestine, amplifying gut permeability and gut–brain inflammatory axis, which links oral dysbiosis to neural dysfunction [28].

A unifying pathway across *P. gingivalis* –associated systemic diseases is the dual effect of immune activation and immune subversion. In CVD, DM, and CRC, *P. gingivalis* activates the NF‐κB and PI3K/AKT signalling cascades, driving local inflammation, endothelial apoptosis and tumour progression [15, 29, 30]. Additionally, NLRP3 inflammasome activation plays a pivotal role in AD and CRC, where immune activation occurs predominantly within the brain structure or haematopoietic‐derived cells, sustaining neuroinflammation and tumour‐promoting inflammation [25, 28, 31]. In parallel, *P. gingivalis* employs sophisticated immune subversion strategies. In CVD, APO, and CRC, *P. gingivalis* impairs macrophage efferocytosis [16], induces dendritic cell senescence [17], and alters uterine and iNKT cell populations [27, 32].

In addition to immune dysregulation, mitochondrial dysfunction and oxidative stress are convergent mechanisms in *P. gingivalis* –mediated systemic diseases. In endothelial cells, *P. gingivalis* infection suppresses mitochondrial Sirtuin‐3, leading to mitochondrial dysfunction, cellular apoptosis and impaired angiogenic repair [14]. Similarly, in APO, *P. gingivalis* infection induces oxidative stress in placental tissues, marked by upregulation of Htra1 and loss of trophoblast density, disrupting spiral artery remodelling even in the absence of overt inflammation [26, 27].

Beyond localised effects, systemic inflammatory amplification characterises diseases such as CVD, DM, and AD. In CVD, *P. gingivalis* infection induces dendritic cell senescence and triggers the release of exosomes enriched with inflammatory mediators, thereby extending vascular and systemic inflammation [17]. In a metabolic context, chronic *P. gingivalis* infection induces low‐grade systemic inflammation marked by elevated circulating cytokines, reduced expression of insulin sensitivity markers, and impaired glucose metabolism in hepatic and adipose tissues [34]. In AD, *P. gingivalis* promotes systemic inflammation and neuroinflammation. Concurrently, it also disrupts gut microbiota composition, resulting in gut dysbiosis and impaired intestinal barrier integrity, while activating NLRP3 inflammasome. This ultimately leads to the dysregulation of the gut–brain axis to promote the development and progression of AD [23, 25, 28].

These pathogenic mechanisms are further amplified by systemic and local metabolic dysregulation. In insulin‐sensitive tissues, *P. gingivalis* disrupts glucose metabolism by interfering with Akt/GSK3β activation, thereby impairing hepatic glycogen synthesis and promoting insulin resistance [20]. Beyond this local interference, *P. gingivalis* exacerbates systemic low‐grade inflammation, which, together with elevated BCAAs, contributes to the downregulation of insulin sensitivity markers and degradation of Akt2, resulting in impaired insulin signalling and systemic hyperglycaemia [34, 36, 37, 38]. In APOs, metabolic alterations occur locally within placental trophoblasts, where internalisation of *P. gingivalis* downregulates glucose transporters GLUT1 and GLUT3, leading to reduced glycolytic flux and impaired trophoblast migration and invasion [22].

These findings suggest that early detection and management of periodontitis may support the systemic health of periodontitis patients. Reducing the local burden of *P. gingivalis* through periodontal care, host modulation, and targeted antimicrobials may limit its systemic dissemination and the associated inflammation. Despite the strong epidemiological and mechanistic links, clinical interventional studies directly linking periodontal treatment to improved systemic outcomes remain limited. Future research should focus on integrated clinical trials to assess whether targeting *P. gingivalis* could influence the initiation/progression of systemic disease and thus support periodontal therapy as a potential preventive strategy against systemic disease.

## Author Contributions

**Emily Ming‐Chieh Lu:** conceptualization, writing – review and editing, supervision, project administration. **Alaa Ahmed Basalim:** writing – original draft, visualization, writing – review and editing.

## Funding

The authors have nothing to report.

## Conflicts of Interest

The authors declare no conflicts of interest.

## Data Availability

Data sharing not applicable to this article as no datasets were generated or analysed during the current study.
